# The disease that bites – an assessment of demographics, clinical characteristics, and treatment practices of patients with snakebite envenoming across Vietnam

**DOI:** 10.7189/jogh.16.04225

**Published:** 2026-06-26

**Authors:** Quy Quoc Bao Truong, Jade Dean Rae, Phuc Thanh Nhan Nguyen, Thi Thu Thuy Nguyen, Van Thang Vo, Jürgen May, Ralf Krumkamp, Thi Anh Thu Dang, Jörg Blessmann, Benno Kreuels

**Affiliations:** 1 Research Group Neglected Diseases and Envenoming, Bernhard Nocht Institute for Tropical Medicine, Hamburg, Germany; 2 Faculty of Public Health, University of Medicine and Pharmacy, Hue University, Hue, Vietnam; 3 Hue City Association of Public Health and Preventive Medicine, Hue, Vietnam; 4 Science-Technology and International Relations Office, University of Medicine and Pharmacy, Hue University, Hue, Vietnam; 5 University Medical Centre Hamburg-Eppendorf, Hamburg, Germany; 6 Department of Infectious Disease Epidemiology, Bernhard Nocht Institute for Tropical Medicine, Hamburg, Germany; 7 German Centre for Infection Research, Hamburg-Borstel-Lübeck-Riems, Germany

## Abstract

**Background:**

Snakebite envenoming presents a significant burden to the health care system in Vietnam. However, data on snakebite-related hospital visits and the availability of antivenom across the country remain scarce. Furthermore, details on clinical presentation, responsible snakes, and treatment practices are lacking.

**Methods:**

In this retrospective, cross-sectional study, we collected data on snakebite-related visits and antivenom availability in major hospitals across 40 provinces from 2018 to 2022 and analysed a subset of patients’ files. We displayed the geographical distribution of snakebite-related visits, available antivenoms, and responsible snake species across Vietnam, described the clinical presentations of envenoming, and assessed the appropriateness of treatment practices.

**Results:**

We recorded 23 877 snakebite-related visits from 2018 to 2022. Antivenom was available in 29 of 62 (46.8%) hospitals, more commonly in the southern provinces. In 2024 patient files, signs of haemotoxic envenoming and neurotoxic envenoming were recorded in 445 (22.0%) and 59 (2.9%) patients, respectively. The most frequently recorded snake genera were *Trimeresurus* and *Naja*. Antivenom was administered to 434 (33.9%) of the 1280 patients from antivenom-equipped hospitals. Ancillary treatments were commonly used: 1076 (53.1%) patients received antibiotics, and 940 (46.4%) received corticosteroids. In-hospital mortality was low at 0.3% and was mainly due to neurotoxic envenoming.

**Conclusions:**

We recorded a considerable number of snakebite-related visits in participating hospitals; however, antivenom availability was limited, and treatment practices were often inappropriate. Improving access to antivenom and updating national guidelines are essential to further reduce morbidity and mortality of snakebite in Vietnam.

Snakebite envenoming remains a significant public health challenge globally. The World Health Organization (WHO) classifies this toxicological emergency, which affects 5.4 million people and results in 81 000 to 138 000 fatalities and 400 000 cases of permanent disability annually, as one of currently 22 neglected tropical diseases [1]. The burden of snakebites is particularly high in populations with limited access to health care and antivenom [2]. A recent study estimated that 242 648 bites occur annually across seven Southeast Asian countries, including Vietnam, making this region one of the world’s snakebite hotspots [3].

Vietnam is home to a diverse snake fauna that poses a significant risk of snakebite [4]. However, no nationwide assessment of this problem has been conducted. As snakebites are not centrally notifiable, the number of snakebites presenting to the health system nationwide remains unclear. Community-based surveys from individual provinces have revealed a substantial number of snakebites [5,6], and the country’s largest referral hospitals previously reported particularly high burden – Bach Mai Hospital in Hanoi treated an average of 450 patients annually between 2008 and 2020 [7], and Cho Ray Hospital in Ho Chi Minh City treated more than 600 patients in 2000 [8].

In northern Vietnam, cobras (*Naja*) are likely the most common culprits, and their venom can cause serious cyto- and neurotoxicity [7]. In the central and southern regions, green pit vipers (*Trimeresurus*) are primarily responsible for most bites, often resulting in blood coagulation disorders in patients [6,8]. The Institute of Vaccines and Medical Biologicals in Vietnam (IVAC) domestically produces two monovalent antivenoms, one against *Trimeresurus albolabris* and the other against *Naja kaouthia* [9]. However, little is known about the distribution and use of these antivenoms across hospitals, as there is no public database on antivenom availability in the country.

Adequate administration of antivenom is equally important as its availability. While underutilisation can lead to unsatisfactory outcomes, overuse may lead to unnecessary complications. Antivenom is crucial, but pain management, infection control, and other ancillary treatments are also essential for successful snakebite management [10]. For instance, the overuse of antibiotics, which can lead to antimicrobial resistance, is a growing concern in snakebite patients in many settings [11]. In Vietnam, official guidelines for managing snakebites were last updated in 2015 and recommend prophylactic antibiotics, which contrast with the WHO guidelines from 2017 [12].

To address the lack of nationwide data, limited information on antivenom distribution, and uncertainties in treatment practices, we assessed the annual number of snakebite-related hospital visits and the availability of antivenom and described the demographic, clinical and treatment details of snakebite patients treated in hospitals across 40 provinces of Vietnam.

## METHODS

### Study design and settings

We conducted this retrospective cross-sectional study to describe the distribution of snakebite envenoming across Vietnam and assess the availability of antivenom nationwide. Due to operational constraints, we were only able to visit 40 of the 63 provinces. In the selection process, we aimed to ensure that all eight geographical regions of Vietnam were adequately represented [13]. Where we could not visit all provinces within a region, we first excluded provinces for which data were already available and selected the remaining provinces based on accessibility [5,7,8].

Within these provinces, we invited all 77 general secondary- and tertiary-level hospitals to participate in the study. As of 1 July 2025, Vietnam reduced the number of its provincial-level units from 63 to 34, including 28 provinces and six centrally governed cities. However, we retained the 63-province system for reporting the study’s results.

### Data collection

We collected data in a two-step procedure from December 2022 to March 2024. First, we asked the participating hospitals to extract the number of snakebite-related visits recorded between January 2018 and December 2022. The hospitals identified these visits using the International Classification of Diseases 10th edition (ICD-10) codes: X20 (contact with venomous snakes and lizards), W59 (bitten or crushed by other reptiles), or T63 (toxic effect of contact with venomous animals) [14]. Before recording the final number of visits, we asked the hospital to cross-check the coded diagnoses against the written discharge diagnoses and remove all non-snakebite visits (*e.g.* centipede bites) from their reports. The hospitals were also asked to report which antivenom they stocked during the mentioned period. All data collected in this step is referred to as ‘snakebite-related visits.’

Second, we asked hospitals to provide a random sample of patient files, representing 25% of snakebite-related visits in 2021 and 2022, for detailed review. The hospital’s staff used the ‘RAND’ function in Microsoft Excel to select a random subset of all files with one of the required ICD-10 codes. If a file was unavailable or from a non-snakebite patient (as confirmed by the study team), it was replaced with the next selected number. Study team members (QQBT, PTNN, and JB) confirmed that the files were from the required time period and contained sufficient detail before extracting date, age, sex, history of the snakebite incident (*e.g.* species, bite situation), symptoms including local (swelling, blisters, necrosis) and neurotoxic (ptosis, coma, dysarthria, dyspnoea, dysphagia, respiratory failure) signs of envenoming, the highest values of international normalised ratio/prothrombin time (INR/PT) and lowest platelet (PLT) count, as well as treatment provided and outcome of each patient. For patients treated with antivenom, we recorded the type of antivenom and the number of vials administered. All data collected in this step is referred to as ‘patients.’

All data were entered into pre-designed collection forms in Microsoft Excel 2021, version 16.95.1 (Microsoft Corporation, Redmond, Washington, USA).

### Data management and analysis

#### Number of snakebite-related visits and antivenom availability

For each hospital, we calculated the total and annual median (MD) number of snakebite-related visits from 2018 to 2022, along with the 25th (Q1) and 75th (Q3) percentiles. We visualised the locations of the participating hospitals by hospital type, antivenom availability, and the median yearly number of visits on maps. We used Mood’s median test to compare the median number of snakebite-related visits between hospitals with and without antivenom.

#### Data from patient files

For all provided patient files, we excluded those lacking information on age, sex, date of visit, a description of the incident, and details on the outcome. We used descriptive statistics to summarise patient demographics, envenoming characteristics, and laboratory results.

We recorded the culprit snakes and graded the severity of envenoming based on laboratory test results and symptoms. For bites by the Viperidae family or *Rhabdophis* genus, we graded patients with an INR<1.2 (or PT<15 seconds) and PLT count ≥100 × 10^9^/L as ‘mild,’ and patients where either the PLT count or both INR/PT results were missing, but the other test indicated ‘mild’ envenoming, as ‘probably mild.’ Patients with an INR≥1.2 (or PT≥15 seconds if INR was not available) or with PLT counts below 100 × 10^9^/L were considered ‘moderate/severe.’ Patients with all laboratory tests missing were not graded. We classified all patients with Elapidae bites who had recorded signs of neurotoxic envenoming as ‘moderate/severe’ and all others as ‘mild.’ If the culprit snake was not mentioned in files, we categorised patients based on laboratory test results and symptoms into ‘haemotoxic signs’ (INR≥1.2, or PT≥15 seconds if INR was not available, or PLT counts below 100 × 10^9^/L), ‘neurotoxic signs,’ ‘local envenoming signs only,’ and ‘no symptoms.’ Patients without any symptoms and laboratory tests documented were categorised as ‘unclear.’

We assessed the appropriateness of antivenom use based on the type of antivenom, the culprit snake, and the severity or syndromic nature of envenoming. Details on ancillary treatments and hospital stay were summarised and compared by hospital antivenom availability, antivenom administration, and severity gradings using descriptive statistics.

All analyses were performed in *R*, version 4.5.1 (R Core Team, Vienna, Austria), including the coin package for Mood’s median test [15]. Map data was obtained from Open Development Vietnam [16].

## RESULTS

### Hospital participation, visits due to snakebites, and the available antivenom

**Figure 1:**
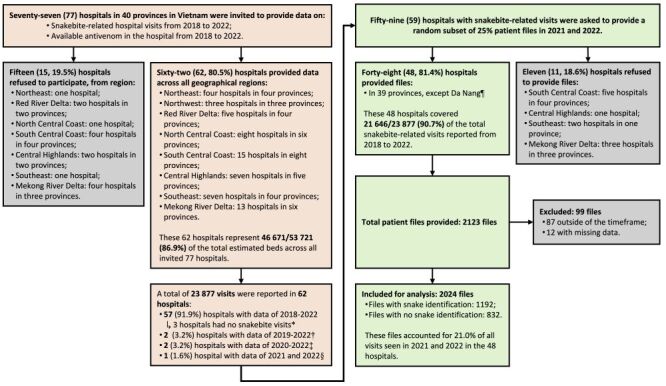
Summary of the two-step procedure of data collection. *Northern Quang Binh Area Hospital, Quang Binh province, Thap Muoi Area Hospital and the Civil Military Hospital, Dong Thap province. †Central An Giang Hospital, An Giang province (294 visits), Long An Provincial Hospital, Long An province (524 visits). ‡Northern Binh Thuan Area Hospital, Binh Thuan province (19 visits), Northern-Mountainous Quang Nam Area Hospital, Quang Nam province (361 visits). §Nam Dinh Provincial Hospital, Nam Dinh province (116 visits). ¶Da Nang General Hospital, Da Nang municipality (710 visits).

Of the 77 hospitals invited, 62 (80.5%) hospitals – accounting for an estimated 46 671/53 721 (86.9%) of the hospital beds at this level of care across 40 provinces – provided data on both the number of snakebite-related visits and the availability of antivenom (**Figure 1**; Table S1 in the **Online Supplementary Document**).

Between 2018 and 2022, 23 877 snakebite-related visits were recorded across 62 hospitals, with an annual MD = 4840 visits, ranging from 4436 in 2018 to 4937 in 2020. The MD total number of visits per hospital from 2018 to 2022 was 281 visits (interquartile range (IQR) = 144–489). The hospital with the highest number of snakebite-related visits was Quang Ngai Provincial Hospital, with 2491 visits (annual MD = 507 visits; IQR = 486–530). Three hospitals reported no visits during the specified timeframe. Overall, the 20 hospitals from 17 northern provinces (24 001 300 inhabitants in 2022) had 3903 visits with a median of 160 visits per hospital (IQR = 126–225), while the 42 hospitals from 23 southern provinces (31 718 700 inhabitants in 2022) reported a total of 19 985 visits (MD = 366; IQR = 175–596).

**Figure 2:**
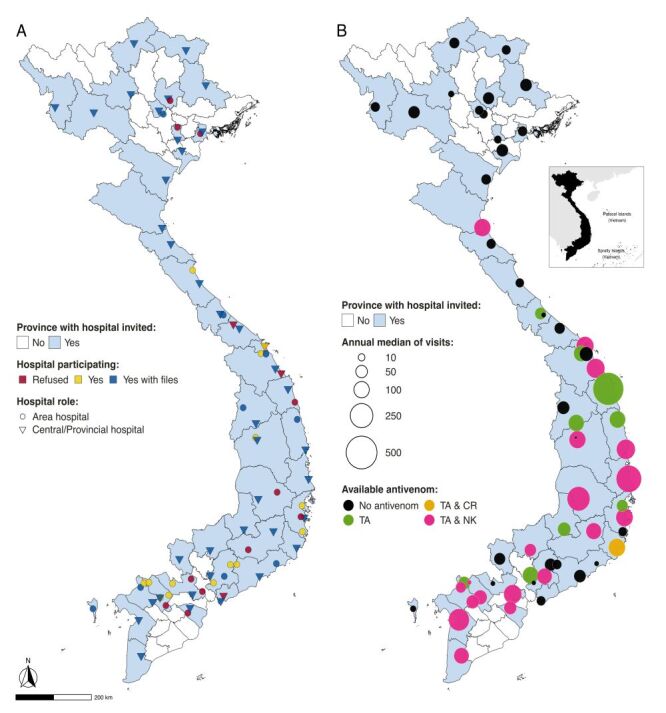
Snakebite-related visits and antivenom availability in hospitals across 40 provinces in Vietnam. **Panel A.** Hospitals’ role and their participation in the study. **Panel B.** The annual median number of snakebite-related hospital visits and the availability of antivenom at hospitals from 2018 to 2022. CR –QSMI C. rhodostoma, NK – IVAC N. kaouthia, TA – IVAC T. albolabris.

During the same period, antivenom was available at 27 (64.3%) of 42 hospitals in the southern provinces, but at only two (10.0%) of 20 hospitals in the country’s northern provinces (**Figure 2**). While IVAC *Trimeresurus albolabris* was available in all 29 antivenom-equipped hospitals, IVAC *Naja kaouthia* was available in only 19 (65.5%). The only imported antivenom, Thailand’s Queen Saovabha Memorial Institute (QSMI) antivenom against *Calloselasma rhodostoma*, was available only at Ninh Thuan Provincial Hospital. In total, antivenom-equipped hospitals had 18 795 visits (MD = 524; IQR = 361–719), and hospitals that did not stock antivenom reported 5092 visits (MD = 148; IQR = 53–213, *P* < 0.001).

### Hospital contribution to patient files

Of the 59 hospitals that reported snakebite-related visits, 48 hospitals provided files of 2123 patients treated in 2021–2022. We excluded 99 (4.7%) files because they fell outside the timeframe (n = 87) or lacked information (n = 12), resulting in 2024 files for analysis.

### Patient demographics, envenoming characteristics, and laboratory results

Of 2024 patients, 61.9% (n = 1252) were male, and 57.0% (n = 1153) worked in agriculture or manual labour. The age was MD = 45 years (IQR = 31–58), and 5.6% (n = 114) of the patients were aged ≤12 years. Most bites were on the arms (n = 966, 47.7%) or legs (n = 933, 46.1%). Local signs of envenoming were observed in 1561 (77.1%) patients, including four patients with venom-induced ophthalmia. Details on the extent of swelling were not available. Signs of haemotoxic envenoming (defined as a pathological INR/PT or PLT<100 × 10^9^/L) were recorded in 445 (22.0%) patients, and signs of neurotoxic envenoming were recorded in 59 (2.9%) patients (**Table 1**).

**Table 1:** Description of snakebite patient files*

Characteristics	n (%)
Hospitalisation days, MD (IQR)	2 (2–4)
Sex	
*Male*	1252 (61.9)
*Female*	772 (38.1)
Age in years	
*Overall, MD (IQR)*	45 (31–58)
*1–12*	114 (5.6)
*13–17*	78 (3.9)
*18–60*	1438 (71.0)
*≥61*	394 (19.5)
Occupation	
*Agricultural/manual worker*	1153 (57.0)
*Other*	871 (43.0)
Envenoming site	
*Upper limb*	966 (47.7)
*Lower limb*	933 (46.1)
*Other*	17 (0.8)
*Not documented*	109 (5.4)
Local envenoming signs*	
*Yes*	1561 (77.1)
*No*	463 (22.9)
Neurotoxic envenoming signs†	
*Yes*	59 (2.9)
*No*	1965 (97.1)
INR test result	
*<1.2*	1105 (54.6)
*≥1.2*	332 (16.4)
*Missing*	587 (29.0)
PT test result in seconds	
*<15*	1217 (60.1)
*≥15*	335 (16.6)
*Missing*	472 (23.3)
PLT count result (× 10^9^/L)‡	
*≥100*	1693 (83.6)
*<100*	125 (6.2)
*Missing*	206 (10.2)
All files reviewed	2024 (100)

INR – international normalised ratio, IQR – interquartile range, MD – median, PLT – platelet, PT – prothrombin time

*Local envenoming: swelling (n = 1553), blisters (n = 40), or tissue necrosis (n = 90).

†Neurotoxic envenoming: ptosis (n = 17), coma (n = 1), dysarthria (n = 4), dyspnoea (n = 19), dysphagia (n = 2), respiratory failure (n = 31).

‡A total of 1191 patients with PLT ≥ 150 × 10^9^/L.

### Identification of the culprit snakes, severity grading, and syndromic groups

**Figure 3:**
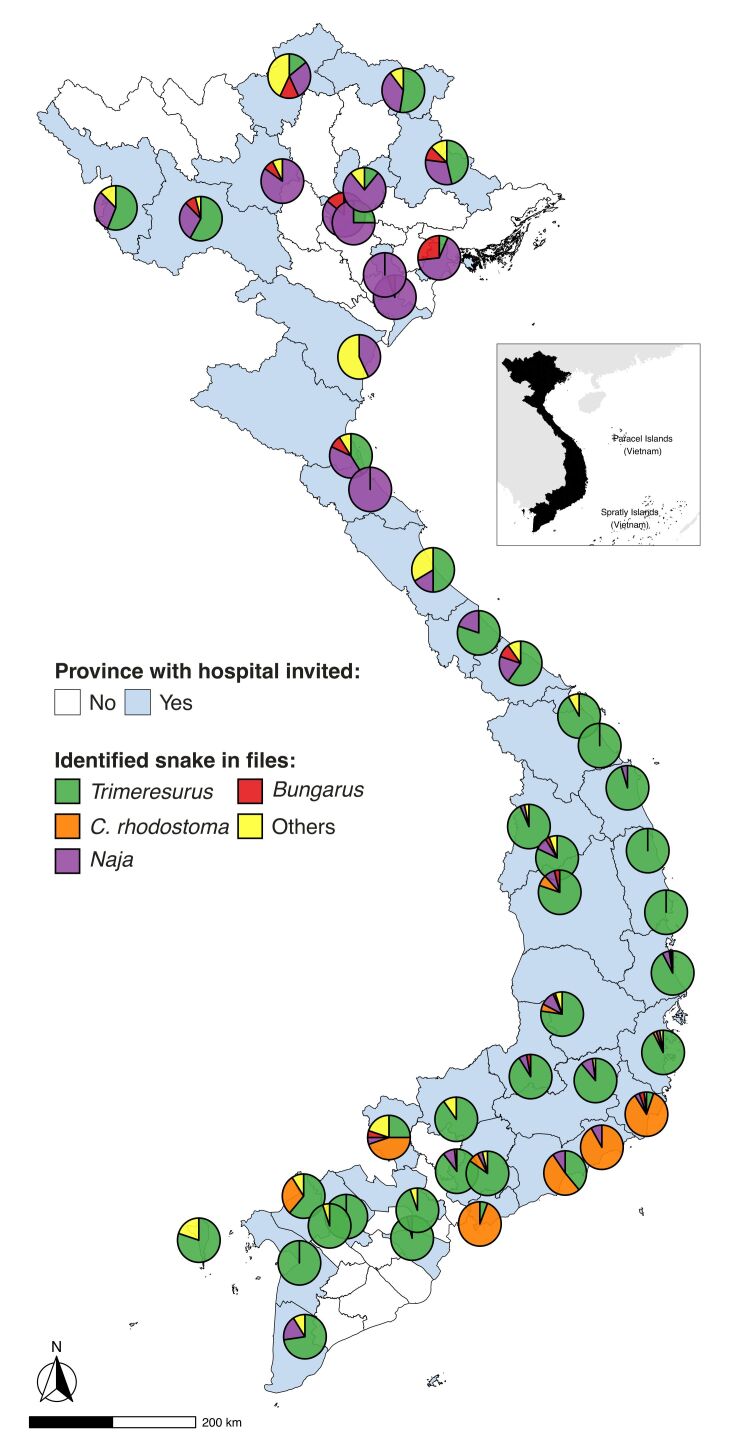
Percentage distribution of 1192 files with identified snakes, from 2024 patient files analysed among 48 hospitals.

A culprit snake was recorded in 1192 (58.9%) patient files (**Figure 3**; Table S2 in the **Online Supplementary Document**). Viperidae, mainly *Trimeresurus* (n = 814) and *Calloselasma rhodostoma* (n = 136), were the most frequently recorded species (**Table 2**). Elapidae were recorded in 199 files, mainly *Naja* (n = 167), followed by *Bungarus* (n = 26). Bites from *Trimeresurus*, *Naja*, and *Bungarus* were seen in hospitals across all regions, while *Calloselasma rhodostoma* bites were seen exclusively in the south (Figure S1 in the **Online Supplementary Document**). Among the 832 patients for whom the culprit snake was not recorded, we were able to assign a systemic envenoming syndrome in 153 (18.4%). Of these, 139/153 (90.8%) were classified as ‘haemotoxic envenoming’ and 14/153 (9.2%) as ‘neurotoxic envenoming’. Among the remaining 679 patients, 390/679 (57.4%) presented with signs of local envenoming, 268/679 (39.5%) showed no symptoms, and 21/679 (3.1%) could not be categorised (**Table 2**).

**Table 2:** Snake identification from patient files with assigned severity grading/syndromic group for each patient, n (%)

Files with identification	Uncategorised severity*		Mild severity group	Probably mild severity group		Moderate/severe group	Total
Family: Viperidae							
*Trimeresurus species (Green pit vipers)*	46 (5.7)		386 (47.4)	127 (15.6)		255 (31.3)	814
*Calloselasma rhodostoma (Malayan pit viper)*	7 (5.1)		76 (55.9)	8 (5.9)		45 (33.1)	136
*Deinagkistrodon acutus (Chinese moccasin)*	0 (0)		0 (0)	0 (0)		2 (100)	2
*Ovophis species (Mountain pit viper)*	0 (0)		0 (0)	0 (0)		2 (100)	2
*Azemiops feae (Fea’s viper)*	0 (0)		1 (100)	0 (0)		0 (0)	1
*Protobothrops species (Brown-spotted pit viper)*	0 (0)		2 (100)	0 (0)		0 (0)	2
Family: Colubridae							
*Rhabdophis species (Red-necked keelbacks)*	0 (0)		2 (33.3)	2 (33.3)		2 (33.3)	6
*Non-venomous or harmless snakes†*	NA		NA	NA		NA	30
Family: Elapidae							
*Naja species (Cobras)*	NA		151 (90.4)	NA		16 (9.6)	167
*Bungarus species (Kraits)*	NA		0 (0)	NA		26 (100)	26
*Ophiophagus hannah (King cobra)*	NA		1 (33.3)	NA		2 (66.7)	3
Sea snake of unknown species	NA		2 (66.7)	NA		1 (33.3)	3
Files without an identification							
*Patients with haemotoxic signs‡*		NA	NA	NA	NA		139
*Patients with neurotoxic signs§*		NA	NA	NA	NA		14
*Patients with signs of local envenoming only¶*		NA	NA	NA	NA		390
*Patients with no symptoms║*		NA	NA	NA	NA		268
Unclear**		NA	NA	NA	NA		21
All files		2024					

NA – not applicable

*Includes Viperidae bites with local signs of envenoming (*Trimeresurus* species, n = 43; *C. rhodostoma*, n = 6) or no symptoms (*Trimeresurus* species, n = 3; *C. rhodostoma*, n = 1), and files with all laboratory tests missing; Elapidae bites are not applicable.

†Includes *Ptyas* species (n = 7), *Coelognathus* species (n = 6), *Oligodon* species (n = 6), *Ahaetulla* species (n = 3), *Fowlea flavipunctata* (n = 3), *Chrysopelea* species (n = 2), *Xenopeltis uniclolor* (n = 2), and *Boiga multomaculata* (n = 1).

‡Possible Viperidae family or *Rhabdophis* genus.

§Possible Elapidae family.

¶Includes 16 (4.4%) patients with all laboratory tests missing; 69 (17.7%) patients with at least one available normal laboratory test, and 305 (78.2%) patients with all normal tests.

║Includes patients with documented absence of local symptoms and at least one available normal laboratory test.

**Patients without any symptoms and laboratory tests documented.

Of the 1192 patients with a recorded culprit snake, we graded 351 (29.4%) as moderate/severe. Of these, 306 (87.1%) exhibited signs of haemotoxic envenoming caused by Viperidae (n = 304) or *Rhabdophis* (n = 2). Among these, the median INR was 1.4 (IQR = 1.3–1.9), the PT was MD = 18 seconds (IQR = 16–30), and the PLT count was MD = 49 × 10^9^/L (IQR = 18–82). The remaining 45 (12.9%) moderate/severe patients were due to neurotoxic envenoming by Elapidae.

### Administration of antivenom

Of the 2024 patients, 1280 (63.2%) were treated in antivenom-equipped hospitals. Of these, 434/1280 (33.9%) patients received antivenom, with 412/434 (94.9%) receiving IVAC *Trimeresurus albolabris* and 22/434 (5.1%) receiving *IVAC Naja kaouthia*. The number of vials administered per patient was MD = 10 (IQR = 5–17) for both antivenoms. The total number of vials used ranged from one to 70 per patient for IVAC *Trimeresurus albolabris* and from one to 35 per patient for IVAC *Naja kaouthia*.

Among the 412 patients who received IVAC *Trimeresurus albolabris* antivenom, 350 (85.0%) patients were attributed to *Trimeresurus*, five (1.2%) to other species, including *Calloselasma rhodostoma* (n = 4) and *Naja* (n = 1), and 57 (13.8%) to unidentified snakes. In the 350 patients attributed to *Trimeresurus*, accounting for 50.9% of the 688 *Trimeresurus* bites treated in antivenom-equipped hospitals, 126/350 (36.0%) patients showed signs of moderate/severe envenoming, suggesting adequate antivenom use. For the remaining 224/350 (64.0%) patients, only mild envenoming signs were recorded, and the indication for antivenom remained unclear. Among the 57 patients who received IVAC *Trimeresurus albolabris* antivenom after bites by unidentified snakes, 18 (31.6%) presented with haemotoxic signs, while the remaining 39 (68.4%) presented only with signs of local envenoming.

Among the 22 patients who received IVAC *Naja kaouthia* antivenom, 13 (59.1%) bites were attributed to *Naja*, one (4.5%) to *Ophiophagus hannah*, and eight (36.4%) to unidentified snakes. Signs of neurotoxic envenoming were not recorded in any of the patients bitten by a *Naja*, and the indication for antivenom remained unclear. Among the eight patients who received IVAC *Naja kaouthia* antivenom after bites by unidentified snakes, only two presented with signs of neurotoxic envenoming, while five showed only local symptoms, and one patient had an unmeasurably high INR and a PLT count of 28 × 10^9^/L. Among these patients, the administration of antivenom was questionable.

### Ancillary treatment practices in all hospitals

A total of 1612 (79.6%) patients received analgesics. Other common medications included antibiotics (n = 1076, 53.1%), corticosteroids (n = 940, 46.4%), and antihistamines (n = 569, 28.1%). Only 808 patients (39.9%) received tetanus prophylaxis. Patients presenting to antivenom-equipped hospitals were more likely to receive ancillary treatment, *e.g.* 88.4% *vs*. 64.7% receiving analgesics (**Table 3**). Within antivenom-equipped hospitals, patients who received antivenom were also more likely to receive ancillary treatments, *e.g.* 60.1% *vs*. 40.8% receiving corticosteroids (Table S3 in the **Online Supplementary Document**).

**Table 3:** Ancillary treatment practices in patients with and without antivenom treatment, n (%)

Treatment	Patients with antivenom treatment in antivenom-equipped hospitals (n = 434)	Patients without antivenom treatment in antivenom-equipped hospitals (n = 846)	Patients in non-antivenom-equipped hospitals (n = 744)	All patients (n = 2024)
Analgesics	413 (95.2)	718 (84.9)	481 (64.7)	1612 (79.6)
Antibiotics	302 (69.6)	401 (47.4)	373 (50.1)	1076 (53.1)
Corticosteroids	261 (60.1)	345 (40.8)	334 (44.9)	940 (46.4)
Tetanus toxoid	197 (45.4)	349 (41.3)	262 (35.2)	808 (39.9)
Antihistamines	166 (38.2)	190 (22.5)	213 (28.6)	569 (28.1)
Fresh frozen plasma	53 (12.2%)	35 (4.1)	24 (3.2)	112 (5.5)
Vitamin K	35 (8.1)	17 (2.0)	42 (5.6)	94 (4.6)
Tranexamic acid	13 (3.0)	6 (0.7)	19 (2.6)	38 (1.9)
Whole blood transfusion	10 (2.3)	7 (0.8)	7 (0.9)	24 (1.2)
Platelet transfusion	4 (0.9)	9 (1.1)	4 (0.5)	17 (0.8)
No treatment documented	0 (0)	22 (2.6)	12 (1.6)	34 (1.7)

### Hospital outcomes

Overall, 1337 (66.1%) patients were discharged by the medical team, 526 (26.0%) opted for self-discharge, 154 (7.6%) were transferred to another hospital, and seven died in-hospital (0.3%). A responsible snake was recorded for 112/154 (72.7%) of transferred patients. The most common species were *Calloselasma rhodostoma* (n = 40), *Naja* (n = 36), *Trimeresurus* (n = 23), and *Bungarus* (n = 7). Of 59 patients involving *Trimeresurus* or *Naja*, 45 (76.3%) were transferred from hospitals lacking respective antivenom for these species. Of all transferred patients, only 80/154 (51.9%) had signs of systemic envenoming (n = 60 haemotoxic, n = 20 neurotoxic).

One (14.3%) of the seven deaths was attributed to *Trimeresurus* and exhibited coagulation disorder; five were attributed to Elapidae (*Naja* n = 3; *Bungarus* n = 2) with signs of neurotoxic envenoming; and one was bitten by an unidentified snake with signs of neurotoxic envenoming, suggesting a possible Elapidae bite. Four of the seven patients died in antivenom-equipped hospitals, and only one was treated with antivenom (Table S4 in the **Online Supplementary Document**).

## DISCUSSION

While it is well known that snakebite envenoming is an endemic neglected tropical disease in Vietnam, we present here the first nationwide assessment of snakebites and antivenom availability in hospitals. With nearly 5000 annual hospital visits, snakebites presented a considerable health burden across Vietnam, while antivenom availability was limited, with only half of the surveyed hospitals stocking this essential drug. Most bites were due to *Trimeresurus*, *Calloselasma rhodostoma*, *Naja*, and *Bungarus*. Treatment practices often seemed inadequate, but in-hospital mortality was low and occurred primarily in patients with neurotoxic envenoming.

While the 23 877 hospital visits identified in this study from 2018 to 2022 represent a considerable number of snakebite cases, the total number in the country is likely significantly higher. We targeted only general secondary- and tertiary-level hospitals in 40 of 63 provinces, and only 62 of the 77 hospitals participated. Therefore, we probably captured less than two-thirds of the snakebite-related visits at this level of care. A study conducted at the largest referral hospital in northern Vietnam reported that this hospital alone saw an additional 2395 bites from 2018 to 2020 [7]. However, as snakebite envenoming is currently not a notifiable disease, it is unlikely that a more complete overview of all snakebite-related hospital visits will be obtained in the near future. Additionally, snakebite victims presenting to hospitals account for only a fraction of those occurring in the community. Two community-based studies in Thua Thien Hue Province and Can Tho Municipality found that only about one-third of snakebite victims sought hospital care [5,6]. Despite our study period spanning the COVID-19 pandemic, we did not observe a clear effect of lockdowns on the number of snakebite-related visits (Table S1 in the **Online Supplementary Document**). This is possibly because snakebite is a medical emergency for which patients continued to access formal care even during lockdowns, though access barriers and care avoidance were reported in many other settings [17].

Hospitals in the southern provinces reported significantly more snakebite-related visits than those in the northern provinces. Despite not being directly assessed in this study, this difference may reflect variations in health care-seeking behaviour. For instance, snakebite victims among the large ethnic minority populations in the Northern midlands and uplands may be less inclined to seek formal medical care due to illiteracy, difficulty in accessing health services, or cultural preferences for traditional healers [13,18]. Moreover, the lack of antivenom in hospitals in the northern provinces might have reduced patients' willingness to seek care at these facilities. In northern Vietnam, Bach Mai Hospital in Hanoi functions as the main referral centre, leading to centralisation of antivenom and expertise [7,19]. This centralisation may have contributed to provincial hospitals not stocking antivenom, lower attendance by snakebite victims, and treatment delays due to longer travel distances. Case studies in African countries have shown that stocking antivenom and improving treatment increase the likelihood that snakebite victims will seek care at health facilities [20], which aligns with our experience in the Lao People’s Democratic Republic, where snakebite-related visits increased dramatically after the introduction of antivenom at a provincial hospital. A modelling study from Brazil has demonstrated that scaling up antivenom provision is both cost-effective for the health care system and cost-saving from a societal perspective [21]. The importance of stocking antivenom is further underpinned by our finding that a significant number of referrals came from hospitals without antivenom. In patients with snakebites, time to treatment is one of the main predictors of clinical outcomes, and such delays can contribute to mortality and disability.

Most antivenom-equipped hospitals stocked domestic products targeting *Naja kaouthia* and *Trimeresurus albolabris*. However, the culprit snakes were often identified only at the genus level (*e.g.* ‘green pit vipers’ for all *Trimeresurus* and ‘cobra’ for all *Naja*). While Thailand’s QSMI neuro-polyvalent and monovalent *Trimeresurus albolabris* antivenoms have shown cross-reactivity within the respective snake genera [22–24], the cross-neutralisation effectiveness of the Vietnamese antivenoms remains unclear. We documented patients involving extraordinary use of antivenom; for example, one patient received up to 70 vials of IVAC *Trimeresurus albolabris* during their hospitalisation. This raises questions about whether the local antivenom was ineffective at neutralising the venom or whether the antivenom stockpile was misused. While misuse is theoretically possible, it seems unlikely as documentation was available for supply by the pharmacy, prescription by the doctor, and application by the nurse. Other venomous snakes were documented in files, including *Bungarus* across the country, *Calloselasma rhodostoma* in several southern provinces, and *Deinagkistrodon acutus* in the northern Ha Giang province; however, efforts to develop domestic antivenoms for these snakes have not progressed to commercial availability [25]. Internationally produced antivenoms against these snakes were unavailable at the hospitals participating in this study, except for QSMI monovalent antivenom against *Calloselasma rhodostoma*, which was available in limited stock at Ninh Thuan Provincial Hospital. International collaboration could be strengthened to facilitate the import of these antivenoms from neighbouring countries, such as Thailand or China [26], ideally with confirmation of neutralisation potency against the Vietnamese snake fauna.

Across antivenom-equipped hospitals, antivenom was frequently administered to patients with mild envenoming, while, paradoxically, it was withheld in several severe cases. The practice of using antivenom without clear indications, emphasised by WHO as ‘inappropriate use’, raises significant concerns, as it exposes patients to risks of adverse reactions and wastes antivenom resources [1]. At the same time, withholding potentially life-saving antivenom when indicated leads to unnecessarily poor patient outcomes. Ancillary treatment practices were characterised by a high rate of prophylactic antibiotic use and significant administration of non-indicated drugs, such as corticosteroids, vitamin K, or tranexamic acid [11,27]. These practices reflect both clinician caution and a lack of up-to-date local guidelines for standardised treatment, as well as poor adherence to international recommendations. While further studies are needed to assess health care workers’ knowledge and understand the reasons behind these common practices, updating and standardising the national snakebite treatment protocols can already be prioritised [28].

The in-hospital mortality rate due to snakebites was low in our study compared with the 8.0% and 2.8% reported in studies from Myanmar (2018) and Thailand (1989), respectively [29,30]. However, the mortality rate in our study may be underestimated due to the substantial number of transferred patients for whom we were unable to determine final outcomes. In addition, a significant proportion of patients were self-discharged, and the outcome here is also unknown, even if the mortality in this group is likely to be low. Out of seven deaths, four occurred at hospitals where antivenom was reported to generally be available during the study period, and only one patient actually received antivenom. This suggests likely inadequate management of snakebite patients. However, this may also have been due to temporary antivenom stockouts at the time, even if not documented in the patient files. Most deaths were associated with neurotoxic envenoming of cobras and kraits, which were comparable to data from Thailand, reiterating the importance of antivenom availability and timely intervention for neurotoxic envenoming [31,32].

While enabling us to provide the most comprehensive overview of snakebite envenoming in Vietnam to date, our study comes with several limitations. First, we visited 40 of 63 provinces, and only 62 of 77 hospitals provided data. As described above, this leads to an underestimation of the total visits. Second, only 48 of the 62 hospitals provided files for analysis. This may have introduced bias into the patterns of responsible snakes and treatment practices observed in the file data. However, we have no reason to assume that the participating hospitals are not representative of those that did not participate. As at least one hospital in each participating province provided data, we maintained geographical representation. Third, due to privacy constraints, we could not verify if any non-snakebite-related visits were included in the total number of visits reported and had to rely on the accuracy of the hospital staff’s work. This may have led to an overestimation of the total number of snakebites. However, when we extracted data from the patient files, obtained based on ICD-10 codes without pre-screening by hospital staff, we rarely found files with other bites. Fourth, snake identification was frequently missing from files; when present, it was often at the genus level and relied on verbal reports or clinicians’ judgment, making it error-prone. However, this is unlikely to significantly impact our findings, as clinical presentation generally matched the presumed species. Misidentification is also unlikely to have been systematic, although a few bites, attributed to venomous snakes, might have been caused by a harmless one (*e.g.* a harmless green snake as a *Trimeresurus*). Finally, clinical data were not systematically recorded. For example, the absence of documented, reliable signs of systemic bleeding might have underestimated haemotoxic complications. However, systemic bleeding was unlikely with normal laboratory results, and given that laboratory tests were rarely missing, patients experiencing severe bleeding would likely have undergone further investigation. Incomplete documentation of swelling progression may also have led to an underestimation of potential indications for antivenom; nonetheless, antivenom was administered to patients where swelling was not recorded at all.

## CONCLUSIONS

In this study, we provided an overview of the burden of snakebite, antivenom availability, clinical symptoms, responsible snakes, and treatment practices in Vietnam. We identified a considerable number of snakebite-related hospital visits, but with significant gaps in management and a lack of antivenom in many hospitals. To reduce morbidity and mortality from snakebite in Vietnam, improving the availability of antivenom for medically relevant snakes is essential. Future studies should also focus on exploring health care workers’ knowledge of common practices and on improving guideline-adherent clinical management.

## Additional material


Online Supplementary Document
