# Supplementary material for: The disease that bites – an assessment of demographics, clinical characteristics, and treatment practices of patients with snakebite envenoming across Vietnam
Source: J Glob Health. 2026 Jun 26;16:04225. doi: 10.7189/jogh.16.04225 (PMC13306317; doi:10.7189/jogh.16.04225)
Supplement: Online Supplementary Document [file jogh-16-04225-s001.pdf]

Supplement to: Truong QQB, Jade R, Nguyen PTN, Nguyen TTT, Vo VT, May J, Krumkamp R, Dang TAT, Blessmann J, Kreuels B. The disease that bites – an assessment of demographics, clinical characteristics, and treatment practices of patients with snakebite envenoming across Vietnam. J Glob Health. 2026;16:04225.

Supplementary Materials

Table of Contents

STROBE statement 2

Table S1. Summary of participating hospitals, number of snakebite-related visits, and contributed patient files for the review 4

Table S2. Summary of the available antivenom reported at hospitals, files contributed with a snake identification, and the number of venomous snakes recorded from the patient files 6

Table S3. Summary of ancillary treatment by files with snake identification 8

Table S4. Description of in-hospital deaths from the files analysis 9

Figure S1. Percentage distribution of all cases identified as (A) *Trimeresurus*, (B) *C. rhodostoma*, (C) *Naja*, and (D) *Bungarus* in individual hospitals to the total number of identified cases of each snake in files. 10

STROBE Statement—Checklist of items that should be included in reports of *cross-sectional studies*

Title: The Disease that Bites–An assessment of demographics, clinical characteristics, and treatment practices of patients with snakebite envenoming across Vietnam

	Item No	Recommendation	Our paper
**Title and abstract**	1	(*a*) Indicate the study's design with a commonly used term in the title or the abstract	See 'Methods'/Abstract
		(*b*) Provide in the abstract an informative and balanced summary of what was done and what was found	See abstract
**Introduction**			
Background/rationale	2	Explain the scientific background and rationale for the investigation being reported	Paragraphs 1-4/'Introduction'
Objectives	3	State specific objectives, including any prespecified hypotheses	Paragraph 5/'Introduction'
**Methods**			
Study design	4	Present key elements of study design early in the paper	'Study design and settings' section
Setting	5	Describe the setting, locations, and relevant dates, including periods of recruitment, exposure, follow-up, and data collection	'Study design and settings' section and paragraphs 1 and 2 of 'Data collection' section
Participants	6	Give the eligibility criteria, and the sources and methods of selection of participants	'Study design and settings' section and paragraphs 1 and 2 of 'Data collection' section
Variables	7	Clearly define all outcomes, exposures, predictors, potential confounders, and effect modifiers. Give diagnostic criteria, if applicable	Paragraphs 1 and 2 of 'Data collection' section and 'Data management and analysis' section
Data sources/ measurement	8*	For each variable of interest, give sources of data and details of methods of assessment (measurement). Describe comparability of assessment methods if there is more than one group	Paragraphs 1 and 2 of 'Data collection' section and 'Data management and analysis' section
Bias	9	Describe any efforts to address potential sources of bias	Paragraph 2, 5, and 6/'Discussion'
Study size	10	Explain how the study size was arrived at	'Study design and settings' section; Figure 1 and 'Hospital contribution to case files' of 'Results' section
Quantitative variables	11	Explain how quantitative variables were handled in the analyses. If applicable, describe which groupings were chosen and why	Paragraphs 1 and 2 of 'Data collection' section and 'Data management and analysis' section
Statistical methods	12	(*a*) Describe all statistical methods, including those used to control for confounding	Paragraph 3 of 'Data collection' section and 'Data management and analysis' section
		(*b*) Describe any methods used to examine subgroups and interactions	Not applicable
		(*c*) Explain how missing data were addressed	Paragraphs 1 and 2 of 'Data collection' section and 'Data management and analysis' section; Figure 1 and 'Hospital contribution to case files' of 'Results' section

		(*d*) If applicable, describe analytical methods taking account of sampling strategy	Not applicable
		(*e*) Describe any sensitivity analyses	Not applicable
**Results**			
Participants	13*	(a) Report numbers of individuals at each stage of study—eg numbers potentially eligible, examined for eligibility, confirmed eligible, included in the study, completing follow-up, and analysed	Figure 1; 'Hospital participation, visits due to snakebites, and the available antivenom' and 'Hospital contribution to case files' sections
		(b) Give reasons for non-participation at each stage	Figure 1; 'Hospital participation, visits due to snakebites, and the available antivenom' section
		(c) Consider use of a flow diagram	Figure 1
Descriptive data	14*	(a) Give characteristics of study participants (eg demographic, clinical, social) and information on exposures and potential confounders	'Hospital participation, visits due to snakebites, and the available antivenom' and 'Patient demographics, envenoming characteristics, and laboratory results' sections; Figure 2 and Table 1
		(b) Indicate number of participants with missing data for each variable of interest	Figures 1, Figure 2, and Table 1
Outcome data	15*	Report numbers of outcome events or summary measures	See 'Results'
Main results	16	(*a*) Give unadjusted estimates and, if applicable, confounder-adjusted estimates and their precision (eg, 95% confidence interval). Make clear which confounders were adjusted for and why they were included	Not applicable
		(*b*) Report category boundaries when continuous variables were categorized	See 'Results'
		(*c*) If relevant, consider translating estimates of relative risk into absolute risk for a meaningful time period	Not applicable
Other analyses	17	Report other analyses done—eg analyses of subgroups and interactions, and sensitivity analyses	Not applicable
**Discussion**			
Key results	18	Summarise key results with reference to study objectives	Paragraph 1/'Discussion'
Limitations	19	Discuss limitations of the study, taking into account sources of potential bias or imprecision. Discuss both direction and magnitude of any potential bias	Paragraph 2, 5, and 6/'Discussion'
Interpretation	20	Give a cautious overall interpretation of results considering objectives, limitations, multiplicity of analyses, results from similar studies, and other relevant evidence	Paragraphs 2-6/'Discussion'
Generalisability	21	Discuss the generalisability (external validity) of the study results	Paragraph 7/'Discussion'
**Other information**			
Funding	22	Give the source of funding and the role of the funders for the present study and, if applicable, for the original study on which the present article is based	'Funding' section

Table S1. Summary of participating hospitals, number of snakebite-related visits, and contributed patient files for the review

Hospital	Province	Region*†	Beds‡	Snakebite-related visits					Total visits reported	Annual median (Q1–Q3)	Files analysed (percentage of cases seen in 2021-2022)
				2018	2019	2020	2021	2022			
An Giang Area Hospital	An Giang	MRD	850	45	41	42	27	49	204	42 (41–45)	75 (98.7%)
Tan Chau Area Hospital	An Giang	MRD	270	92	69	63	14	37	275	63 (37–69)	No file review
Central An Giang Hospital	An Giang	MRD	950	NA	65	66	73	90	294	70 (66–77)	31 (19.0%)
Vung Tau Hospital	Ba Ria–Vung Tau	SE	420	47	28	36	24	30	165	30 (28–36)	41 (75.9%)
Nguyen Dinh Chieu Hospital	Ben Tre	MRD	1290	39	62	67	202	113	483	67 (62–113)	38 (12.1%)
Bong Son Area Hospital	Binh Dinh	SCC	450	25	129	119	157	59	489	119 (59–129)	49 (22.7%)
Binh Dinh Provincial Hospital	Binh Dinh	SCC	1050	214	236	145	171	168	934	171 (168–214)	63 (18.6%)
Binh Phuoc Provincial Hospital	Binh Phuoc	SE	600	84	55	58	71	38	306	58 (55–71)	14 (12.8%)
Northern Binh Thuan Area Hospital	Binh Thuan	SCC	250	NA	NA	4	5	10	19	5 (4–8)	15 (100%)
Southern Binh Thuan Area Hospital	Binh Thuan	SCC	320	31	43	31	12	27	144	31 (27–31)	No file review
Binh Thuan Provincial Hospital	Binh Thuan	SCC	910	72	77	60	55	38	302	60 (55–72)	61 (65.6%)
Ca Mau Provincial Hospital	Ca Mau	MRD	500	58	135	109	61	104	467	104 (61–109)	38 (23.0%)
Cao Bang Provincial Hospital	Cao Bang	NE	550	30	35	25	30	33	153	30 (30–33)	41 (65.1%)
Danang General Hospital	Da Nang city	SCC	1900	170	197	145	123	75	710	145 (123–170)	No file review
Central Highlands Regional Hospital	Dak Lak	CH	800	135	259	304	271	266	1235	266 (259–271)	100 (18.6%)
Dak Nong Provincial Hospital	Dak Nong	CH	400	53	74	44	122	78	371	74 (53–78)	40 (20.0%)
Dien Bien Provincial Hospital	Dien Bien	NW	650	43	37	32	31	19	162	32 (31–37)	41 (82.0%)
Long Thanh Area Hospital	Dong Nai	SE	510	6	3	9	4	4	26	4 (4–6)	No file review
Dinh Quan Area Hospital	Dong Nai	SE	490	53	61	57	41	64	276	57 (53–61)	No file review
Long Khanh Area Hospital	Dong Nai	SE	600	105	78	102	90	113	488	102 (90–105)	49 (24.1%)
Dong Nai Provincial Hospital	Dong Nai	SE	1100	112	129	121	103	122	587	121 (112–122)	71 (31.6%)
Thap Muoi Area Hospital	Dong Thap	MRD	140	0	0	0	0	0	0	NA	NA
Civil Military Hospital	Dong Thap	MRD	167	0	0	0	0	0	0	NA	NA
Hong Ngu Area Hospital	Dong Thap	MRD	260	6	4	6	4	2	22	4 (4–6)	No file review
Dong Thap Provincial Hospital	Dong Thap	MRD	700	82	97	77	82	120	458	82 (82–97)	52 (25.7%)
331 Hospital	Gia Lai	CH	120	1	1	1	1	1	5	1 (1–1)	No file review
Gia Lai Provincial Hospital	Gia Lai	CH	800	140	135	129	121	112	637	129 (121–135)	41 (17.6%)
Ha Giang Provincial Hospital	Ha Giang	NE	500	41	25	50	37	43	196	41 (37–43)	39 (48.8%)
Ha Tinh Provincial Hospital	Ha Tinh	NCC	1000	33	28	39	35	9	144	33 (28–35)	12 (27.3%)
Viet Tiep Friendship Hospital	Hai Phong city	RRD	1572	35	34	32	33	46	180	34 (33–35)	31 (39.2%)
Hung Yen Provincial Hospital	Hung Yen	RRD	500	14	28	17	19	36	114	19 (17–28)	41 (74.5%)
Cam Ranh Area Hospital	Khanh Hoa	SCC	235	42	36	17	26	27	148	27 (26–36)	No file review
Ninh Hoa Area Hospital	Khanh Hoa	SCC	250	56	59	34	53	48	250	53 (48–56)	No file review
Khanh Hoa Provincial Hospital	Khanh Hoa	SCC	1250	163	134	138	110	156	701	138 (134–156)	61 (22.9%)
Phu Quoc Health Centre	Kien Giang	MRD	400	19	6	6	11	11	53	11 (6–11)	8 (36.4%)
Kien Giang Provincial Hospital	Kien Giang	MRD	1200	214	183	191	237	262	1087	214 (191–237)	23 (4.6%)
Ngoc Hoi Area Hospital	Kon Tum	CH	170	67	55	73	64	45	304	64 (55–67)	44 (40.4%)

Kon Tum Provincial Hospital	Kon Tum	CH	750	115	128	140	103	58	544	115 (103–128)	58 (36.0%)
Lam Dong Provincial Hospital	Lam Dong	CH	590	90	109	145	149	106	599	109 (106–145)	64 (25.1%)
Lang Son Provincial Hospital	Lang Son	NE	800	69	56	69	59	44	297	59 (56–69)	63 (61.2%)
Dong Thap Muoi Area Hospital	Long An	MRD	500	6	5	4	7	2	24	5 (4–6)	No file review
Long An Provincial Hospital	Long An	MRD	930	NA	44	173	155	152	524	154 (125–160)	49 (16.0%)
Nam Dinh Provincial Hospital	Nam Dinh	RRD	600	NA	NA	NA	61	55	116	58 (56–60)	26 (22.4%)
Nghe An Friendship Hospital	Nghe An	NCC	700	122	130	123	141	203	719	130 (123–141)	62 (18.0%)
Ninh Thuan Provincial Hospital	Ninh Thuan	SCC	1000	116	125	134	170	220	765	134 (125–170)	91 (23.3%)
Phu Yen Provincial Hospital	Phu Yen	SCC	800	333	383	376	306	291	1689	333 (306–376)	98 (16.4%)
Northern Quang Binh Area Hospital	Quang Binh	NCC	500	0	0	0	0	0	0	NA	NA
Vietnam Cuba Friendship Hospital	Quang Binh	NCC	944	21	25	27	27	31	131	27 (25–27)	11 (19.0%)
Quang Nam Area Hospital	Quang Nam	SCC	1040	122	73	86	61	79	421	79 (73–86)	29 (20.7%)
Northern-Mountainous Quang Nam Area Hospital	Quang Nam	SCC	1500	NA	NA	164	96	101	361	101 (98–132)	No file review
Quang Nam Provincial Hospital	Quang Nam	SCC	1000	163	173	158	161	164	819	163 (161–164)	46 (14.2%)
Quang Ngai Provincial Hospital	Quang Ngai	SCC	900	537	530	486	431	507	2491	507 (486–530)	56 (6.0%)
Trieu Hai Area Hospital	Quang Tri	NCC	200	16	12	0	5	1	34	5 (1–12)	5 (83.3%)
Quang Tri Provincial Hospital	Quang Tri	NCC	850	75	61	53	48	49	286	53 (49–61)	11 (11.3%)
Son La Provincial Hospital	Son La	NW	550	57	86	105	50	35	333	57 (50–86)	39 (45.9%)
Tay Ninh Provincial Hospital	Tay Ninh	SE	700	52	75	53	43	85	308	53 (52–75)	48 (37.5%)
Thai Nguyen Central Hospital	Thai Nguyen	NE	1300	62	56	54	41	46	259	54 (46–56)	24 (27.6%)
Thanh Hoa Provincial Hospital	Thanh Hoa	NCC	1200	34	42	32	66	37	211	37 (34–42)	25 (24.3%)
Hue Central Hospital	Thua Thien Hue	NCC	3343	44	48	40	45	36	213	44 (40–45)	29 (35.8%)
Phuc Yen Area Hospital	Vinh Phuc	RRD	500	41	25	37	9	17	129	25 (17–37)	5 (19.2%)
Vinh Phuc Provincial Hospital	Vinh Phuc	RRD	850	27	23	25	39	44	158	27 (25–39)	31 (37.3%)
Yen Bai Provincial Hospital	Yen Bai	NW	500	7	11	4	23	22	67	11 (7–22)	35 (77.8%)

*CH–Central Highlands, NA–Not applicable, NCC–North Central Coast, NE–Northeast, NW–Northwest, MRD–Mekong River Delta, RRD–Red River Delta, SCC–South Central Coast, SE–Southeast.

†NE, NW, RRD, and NCC are grouped as the northern part, while SCC, CH, SE and MRD are grouped as the southern part of Vietnam.

‡Estimated number of beds reported by hospitals or based on publicly available data-

Table S2. Summary of the available antivenom reported at hospitals, files contributed with a snake identification, and the number of venomous snakes recorded from the patient files

Hospital	Reported available antivenom in hospital from 2018-2022	Files with a snake identification per total files analysed in each hospital	Recorded venomous snake species from patient files										
			GV	MM	CM	MV	FV	HA	RK	CO	KB	KC	SS
An Giang Area Hospital	TA; NK	40/75	27	13	-	-	-	-	-	-	-	-	-
Tan Chau Area Hospital	TA (2019, 2020)	No file review	NA	NA	NA	NA	NA	NA	NA	NA	NA	NA	NA
Central An Giang Hospital	TA; NK	19/31	18	-	-	-	-	-	-	-	-	-	-
Vung Tau Hospital	No antivenom	34/41	2	32	-	-	-	-	-	-	-	-	-
Nguyen Dinh Chieu Hospital	TA; NK	25/38	24	-	-	-	-	-	-	1	-	-	-
Bong Son Area Hospital	TA	39/49	39	-	-	-	-	-	-	-	-	-	-
Binh Dinh Provincial Hospital	TA; NK	52/63	52	-	-	-	-	-	-	-	-	-	-
Binh Phuoc Provincial Hospital	TA; NK	10/14	9	-	-	-	-	-	-	-	-	-	-
Northern Binh Thuan Area Hospital	No antivenom	12/15	-	11	-	-	-	-	-	1	-	-	-
Southern Binh Thuan Area Hospital	No antivenom	No file review	NA	NA	NA	NA	NA	NA	NA	NA	NA	NA	NA
Binh Thuan Provincial Hospital	No antivenom	31/61	12	16	-	-	-	-	-	3	-	-	-
Ca Mau Provincial Hospital	TA; NK	11/38	8	-	-	-	-	-	-	2	-	-	-
Cao Bang Provincial Hospital	No antivenom	19/41	10	-	-	-	1	-	-	7	-	-	-
Danang General Hospital	TA; NK	No file review	NA	NA	NA	NA	NA	NA	NA	NA	NA	NA	NA
Central Highlands Regional Hospital	TA; NK	57/100	44	3	-	-	-	-	-	6	1	2	-
Dak Nong Provincial Hospital	TA (2019-2022)	33/40	30	-	-	-	-	-	-	2	1	-	-
Dien Bien Provincial Hospital	No antivenom	16/41	9	-	-	-	-	-	-	5	-	-	-
Long Thanh Area Hospital	No antivenom	No file review	NA	NA	NA	NA	NA	NA	NA	NA	NA	NA	NA
Dinh Quan Area Hospital	No antivenom	No file review	NA	NA	NA	NA	NA	NA	NA	NA	NA	NA	NA
Long Khanh Area Hospital	TA; NK	26/49	22	2	-	-	-	1	-	1	-	-	-
Dong Nai Provincial Hospital	TA	56/71	50	-	-	-	-	-	-	5	1	-	-
Thap Muoi Area Hospital	No antivenom	No snakebite admission	NA	NA	NA	NA	NA	NA	NA	NA	NA	NA	NA
Civil Military Hospital	No antivenom	No snakebite admission	NA	NA	NA	NA	NA	NA	NA	NA	NA	NA	NA
Hong Ngu Area Hospital	TA; NK	No file review	NA	NA	NA	NA	NA	NA	NA	NA	NA	NA	NA
Dong Thap Provincial Hospital	TA; NK	48/52	48	-	-	-	-	-	-	-	-	-	-
331 Hospital	No antivenom	No file review	NA	NA	NA	NA	NA	NA	NA	NA	NA	NA	NA
Gia Lai Provincial Hospital	TA; NK	25/41	20	2	-	-	-	-	-	2	1	-	-
Ha Giang Provincial Hospital	No antivenom	7/39	1	-	2	1	-	-	-	2	1	-	-
Ha Tinh Provincial Hospital	No antivenom	3/12	-	-	-	-	-	-	-	3	-	-	-
Viet Tiep Friendship Hospital	No antivenom	15/31	1	-	-	-	-	-	-	10	4	-	-
Hung Yen Provincial Hospital	No antivenom	20/41	-	-	-	-	-	-	-	20	-	-	-
Cam Ranh Area Hospital	No antivenom	No file review	NA	NA	NA	NA	NA	NA	NA	NA	NA	NA	NA

Ninh Hoa Area Hospital	No antivenom	No file review	NA	NA	NA	NA	NA	NA	NA	NA	NA	NA	NA
Khanh Hoa Provincial Hospital	TA; NK	39/61	36	1	-	-	-	-	-	1	-	-	-
Phu Quoc Health Centre	No antivenom	5/8	4	-	-	-	-	-	-	-	-	-	1
Kien Giang Provincial Hospital	TA; NK	17/23	17	-	-	-	-	-	-	-	-	-	-
Ngoc Hoi Area Hospital	No antivenom	30/44	28	-	-	-	-	-	-	1	-	-	-
Kon Tum Provincial Hospital	TA	33/58	27	-	-	-	-	-	1	3	1	-	-
Lam Dong Provincial Hospital	TA; NK	44/64	39	-	-	-	-	-	1	4	-	-	-
Lang Son Provincial Hospital	No antivenom	39/63	18	-	-	1	-	-	-	12	4	-	-
Dong Thap Muoi Area Hospital	No antivenom	No file review	NA	NA	NA	NA	NA	NA	NA	NA	NA	NA	NA
Long An Provincial Hospital	TA; NK	18/49	17	-	-	-	-	1	-	-	-	-	-
Nam Dinh Provincial Hospital	No antivenom	18/26	-	-	-	-	-	-	-	17	1	-	-
Nghe An Friendship Hospital	No antivenom	22/62	9	-	-	-	-	-	-	9	2	-	1
Ninh Thuan Provincial Hospital	TA; CR	55/91	3	47	-	-	-	-	-	2	2	-	-
Phu Yen Provincial Hospital	TA; NK	76/98	70	-	-	-	-	-	-	4	1	-	1
Northern Quang Binh Area Hospital	No antivenom	No snakebite admission	NA	NA	NA	NA	NA	NA	NA	NA	NA	NA	NA
Vietnam Cuba Friendship Hospital	No antivenom	6/11	3	-	-	-	-	-	-	1	-	-	-
Quang Nam Area Hospital	No antivenom	12/29	11	-	-	-	-	-	1	-	-	-	-
Northern-Mountainous Quang Nam Area Hospital	No antivenom	No file review	NA	NA	NA	NA	NA	NA	NA	NA	NA	NA	NA
Quang Nam Provincial Hospital	TA; NK (2019-2022)	35/46	35	-	-	-	-	-	-	-	-	-	-
Quang Ngai Provincial Hospital	TA	42/56	40	-	-	-	-	-	-	2	-	-	-
Trieu Hai Area Hospital	No antivenom	0/5	-	-	-	-	-	-	-	-	-	-	-
Quang Tri Provincial Hospital	TA	5/11	4	-	-	-	-	-	-	1	-	-	-
Son La Provincial Hospital	No antivenom	24/39	14	-	-	-	-	-	-	7	2	1	-
Tay Ninh Provincial Hospital	No antivenom	20/48	5	9	-	-	-	-	-	1	1	-	-
Thai Nguyen Central Hospital	No antivenom	9/24	1	-	-	-	-	-	1	7	-	-	-
Thanh Hoa Provincial Hospital	No antivenom	7/25	-	-	-	-	-	-	1	3	-	-	-
Hue Central Hospital	No antivenom	10/29	6	-	-	-	-	-	1	2	1	-	-
Phuc Yen Area Hospital	No antivenom	4/5	1	-	-	-	-	-	-	3	-	-	-
Vinh Phuc Provincial Hospital	No antivenom	7/31	-	-	-	-	-	-	-	6	1	-	-
Yen Bai Provincial Hospital	No antivenom	13/35	-	-	-	-	-	-	-	11	1	-	-
**Total**		**1,192/2,024**	**814**	**136**	**2**	**2**	**1**	**2**	**6**	**167**	**26**	**3**	**3**

CM–*Deinagkistrodon acutus*, CO–*Naja* sp., CR–QSMI *C. rhodostoma*, FV–*Azemiops feae*, GV–*Trimeresurus* sp., HA–*Protobothrops* sp., KB–*Bungarus* sp.; KC–*Ophiophagus hannah*, MM–*Calloselasma rhodostoma*, MV–*Ovophis* sp., NA—Not applicable, NK–IVAC *Naja kaouthia,* RK–*Rhabdophis* sp., TA–IVAC *T. albolabris*, SS–Sea snake of unknown species.

Table S3. Summary of ancillary treatment by files with snake identification

Category	Files with snake identification					Files without snake identification				Overall, N=2,024
	Mild, n=621	Probably mild, n=137	Moderate/Severe, n=351	Non-venomous/harmless snakes, n=30	Not categorised, n=53	Haemotoxic signs, n=139	Neurotoxic signs, n=14	Signs of local envenoming only, n=390	No symptom shown, n=289	
Analgesics*	523 (84.2%)	116 (84.7%)	318 (90.6%)	14 (46.7%)	45 (84.9%)	110 (79.1%)	9 (62.3%)	310 (79.5%)	166 (57.4%)	1,612 (79.6%)
Antibiotics	356 (57.3%)	94 (68.6%)	221 (63.0%)	5 (16.7%)	32 (60.4%)	79 (56.8%)	9 (64.3%)	220 (56.4%)	60 (20.8%)	1,076 (53.1%)
Corticosteroids	318 (51.2%)	54 (39.4%)	209 (59.5%)	5 (16.7%)	14 (26.4%)	79 (56.8%)	7 (50.0%)	194 (49.7%)	60 (20.8%)	940 (46.4%)
Anti-histamin	181 (29.1%)	39 (28.5%)	126 (35.9%)	8 (26.7%)	9 (17.0%)	41 (29.5%)	3 (21.4%)	114 (29.2%)	48 (16.6%)	569 (28.1%)
Tetanus toxoid	301 (48.5%)	37 (27.0%)	144 (41.0%)	8 (26.7%)	12 (22.6%)	50 (36.0%)	2 (14.3%)	148 (37.9%)	106 (36.7%)	808 (39.9%)
Antifibrinolytic	13 (2.1%)	4 (2.9%)	59 (16.8%)	0 (0.0%)	1 (1.9%)	29 (20.9%)	2 (14.3%)	7 (1.8%)	2 (0.7%)	117 (5.8%)
Fresh frozen plasma	8 (1.3%)	10 (7.3%)	64 (18.2%)	0 (0.0%)	1 (1.9%)	21 (15.1%)	0 (0.0%)	8 (2.1%)	0 (0.0%)	112 (5.5%)
Platelet transfusion	1 (0.2%)	0 (0.0%)	13 (3.7%)	0 (0.0%)	0 (0.0%)	2 (1.4%)	0 (0.0%)	1 (0.3%)	0 (0.0%)	17 (0.8%)
Blood transfusion	1 (0.2%)	0 (0.0%)	14 (4.0%)	0 (0.0%)	0 (0.0%)	7 (5.0%)	0 (0.0%)	2 (0.5%)	0 (0.0%)	24 (1.2%)
No treatment documented	3 (0.5%)	2 (1.5%)	0 (0.0%)	3 (10.0%)	2 (3.8%)	2 (1.4%)	0 (0.0%)	4 (1.0%)	18 (6.2%)	34 (1.7%)

*A total of 148 cases were associated with nonsteroidal anti-inflammatory drugs (NSAIDs)

Table S4. Description of in-hospital deaths from the files analysis

Sex	Age	Description in case files	Signs of local envenoming	Signs of neurotoxic envenoming	PT (sec)	INR	PLT (x 10^9/L)	Snake identification in file	Hospitalisation duration (days)	Antivenom use	Available antivenom at hospital†
Male	4	Got bitten by a krait (*Bungarus* sp.) three hours before the admission, the patient was on respiratory failure when being brought to the hospital	No	Yes	15.9	1.3	251	Krait (*Bungarus* sp.)	1	No	TA; NK
Male	8	Got bitten by a snake on left-chest area, swelling and pain developed at bite site with fatigue and hardness of breath	Yes	Yes	NA	NA	32	Unknown snake	2	No	TA; NK
Male	40	Got bitten by a green pit viper at right-ankle area about two and a half hours before coming to the hospital, the patient was on coma when being brought to the hospital	Yes	No	33.6	2.6	108	Green pit viper (*Trimeresurus* sp.)	13	IVAC T. *albolabris* (23 vials)*	TA; NK
Male	45	Patient was bitten by a 4kg cobra, *Naja* sp., with recorded signs of neurotoxic envenoming	Yes	Yes	11.8	1.1	282	Cobra (*Naja* sp.)	1	No	TA; NK
Male	47	Got bitten by a Krait (*Bungarus* sp.) multiple time at upper left-hand area and finger III on the left at two hours before coming to the hospital, the patient was on respiratory and circulation failure when being brought to the hospital	No	Yes	13.9	1.2	2.14	Krait (*Bungarus* sp.)	2	No	No
Male	77	Got bitten by a cobra (*Naja* sp.) at finger III on the right hand at three hours before coming to the hospital, suspected for a *Naja kaouthia* because of the snake's black skin. Swelling moved to hand with pain. Ptosis with dysarthria developed, two arms were weakened at the time	Yes	Yes	NA	NA	101	Cobra (*Naja* sp.)	15	No	No
Male	92	Got bitten by a cobra (*Naja* sp.) at right heel at 30 min before coming to the hospital, no local symtom developed yet, however, developed nerotoxic symtoms, including fatigue and had to be on intubator, with respiratory failure on second hour, was also considered an anaphylactic shock due to snake venom	No	Yes	12.4	1.1	297	Cobra (*Naja* sp.)	1	No	No

INR–International Normalised Ratio, NK–IVAC *N. kaouthia,* PLT – platelet, PT–Prothrombin Time, TA–IVAC *T. albolabris.*
*Total vials given to the patient during hospitalisation.
†Available antivenom at the hospitals reported from 2018-2022.

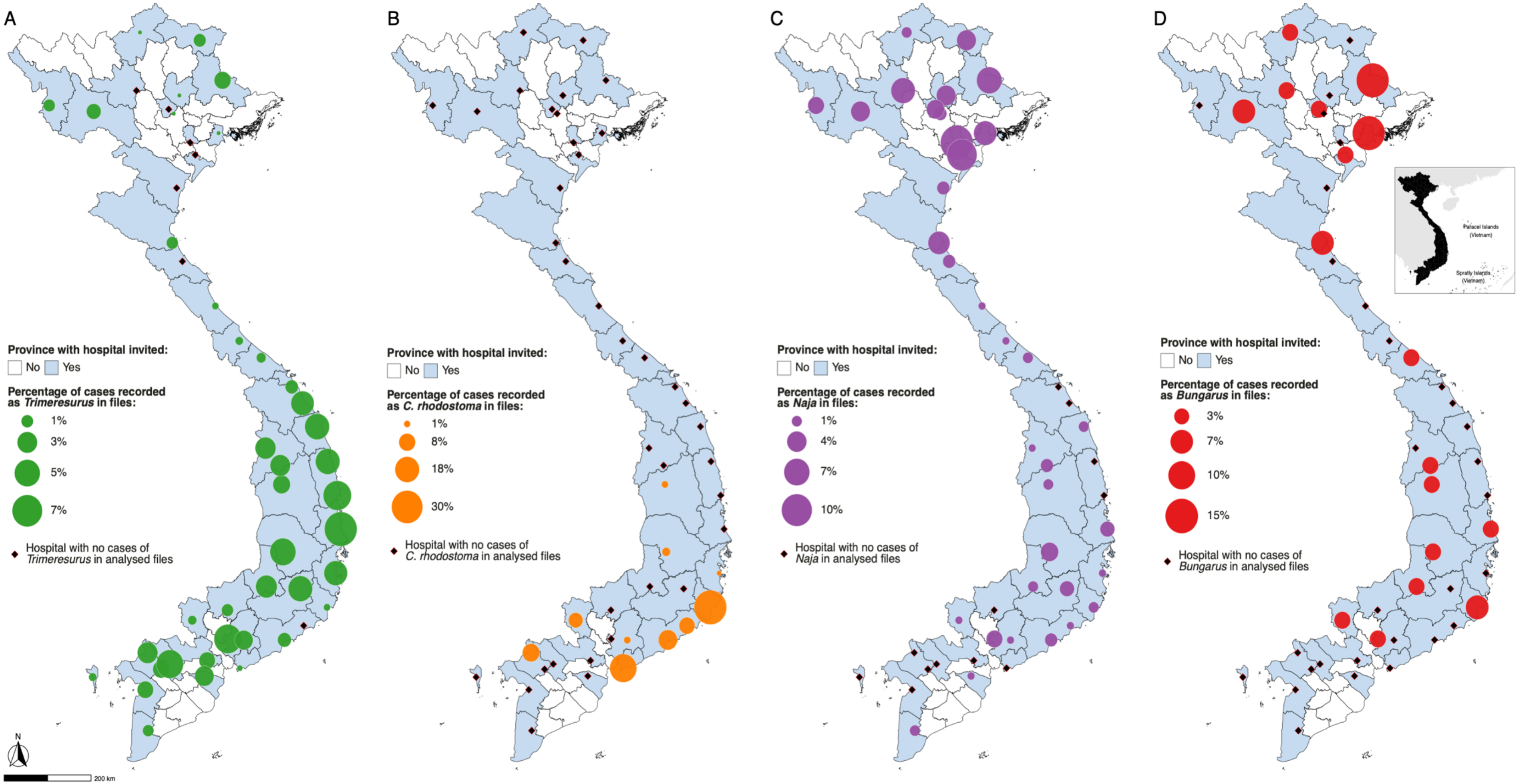

Figure S1. Percentage distribution of all files with a snake identified as (A) *Trimeresurus*, (B) *C. rhodostoma*, (C) *Naja*, and (D) *Bungarus* in individual hospitals to the total number of identified cases of each snake in files.
